# Seed endophytic bacterial profiling from wheat varieties of contrasting heat sensitivity

**DOI:** 10.3389/fpls.2023.1101818

**Published:** 2023-04-06

**Authors:** Krishnan Aswini, Archna Suman, Pushpendra Sharma, Pradeep Kumar Singh, Shrikant Gond, Devashish Pathak

**Affiliations:** ^1^ Division of Microbiology, ICAR-Indian Agricultural Research Institute, New Delhi, India; ^2^ Division of Genetics, ICAR-Indian Agricultural Research Institute, New Delhi, India

**Keywords:** heat sensitivity, seed microbiome, culturable, tolerance, metagenomics, bacterial diversity

## Abstract

Wheat yield can be limited by many biotic and abiotic factors. Heat stress at the grain filling stage is a factor that reduces wheat production tremendously. The potential role of endophytic microorganisms in mitigating plant stress through various biomolecules like enzymes and growth hormones and also by improving plant nutrition has led to a more in-depth exploration of the plant microbiome for such functions. Hence, we devised this study to investigate the abundance and diversity of wheat seed endophytic bacteria (WSEB) from heat^S^ (heat susceptible, GW322) and heat^T^ (heat tolerant, HD3298 and HD3271) varieties by culturable and unculturable approaches. The results evidenced that the culturable diversity was higher in the heat^S^ variety than in the heat^T^ variety and *Bacillus* was found to be dominant among the 10 different bacterial genera identified. Though the WSEB population was higher in the heat^S^ variety, a greater number of isolates from the heat^T^ variety showed tolerance to higher temperatures (up to 55°C) along with PGP activities such as indole acetic acid (IAA) production and nutrient acquisition. Additionally, the metagenomic analysis of seed microbiota unveiled higher bacterial diversity, with a predominance of the phyla Proteobacteria covering >50% of OTUs, followed by Firmicutes and Actinobacteria. There were considerable variations in the abundance and diversity between heat sensitivity contrasting varieties, where notably more thermophilic bacterial OTUs were observed in the heat^T^ samples, which could be attributed to conferring tolerance against heat stress. Furthermore, exploring the functional characteristics of culturable and unculturable microbiomes would provide more comprehensive information on improving plant growth and productivity for sustainable agriculture.

## 1 Introduction

India, being enriched with diverse agro-ecological environments, is the second largest producer of wheat (*Triticum aestivum* L.) worldwide (MoA & FW, 2019), which is cultivated during the Rabi season (Ramadas et al., 2020). It requires an optimal mean temperature ranging from 20 to 25°C (Tripathi and Mishra, 2017). However, the average global temperature is reported to be increasing at a rate of 0.18°C every decade (Hansen et al., 2012). Worldwide, a significant part of wheat areas are experiencing heat stress, and it is estimated that the production of wheat will decrease by 4–6 million tons for every 1°C rise in temperature. There will be a 9%–25% profit loss for rainfed wheat when the temperature rises by 2–3.5°C (Aggarwal et al., 2009; Mukherjee et al., 2019). Several studies have recorded the remarkable influence of high temperatures on wheat (Satorre and Slafer, 1999) and also studied the impact of growing season temperatures in major wheat-producing regions (Asseng et al., 2015; Liu et al., 2016; Hatfield and Dold, 2018; Demirhan, 2020). In 2021, every month experienced warmer temperatures than the average, as stated in the 2021 Global Climate Report (NOAA, National Centers for Environmental Information, 2021) and there was a 15%–20% yield loss observed by CIMMYT’s on-farm experiments in 2022 (Bentley et al., 2022).

Wheat yield, corresponding grain size, and weight will be affected due to intense temperature changes during the sensitive growth stages of flowering, anthesis, and milking (Talukder et al., 2014). In order to accommodate the adverse physiological and biochemical changes that occur due to stress factors, wheat improvement approaches have been proposed to bridge the yield gaps and improve productivity by developing climate-resilient wheat genotypes (Kumar et al., 2019). Several management strategies including changes in agronomic practices such as sowing time, irrigation pattern, nutrient management, timely weeding, and resistant cultivars have been suggested (Singh et al., 2017). Nowadays, genetic engineering and manipulation by molecular breeding are being explored to provide tolerance to several biotic and abiotic stresses, as they offer a more reliable solution than conventional breeding (Varshney et al., 2011).

Currently, the plant microbiome has gained attention just like the human microbiome in maintaining homeostasis (Hirt, 2020; Wagner, 2022) under normal and stress conditions. Characterizing the core microbial communities of the plant and identifying their role are critical inputs in alleviating various stress factors. The modern breakthrough in sequencing technologies has facilitated extensive investigations on microbial communities in the plant vicinity such as the rhizosphere, phyllosphere, and endosphere of important crops and model plant species (Redford and Fierer, 2009; Bulgarelli et al., 2012; Lebeis et al., 2012; Bodenhausen et al., 2013; Shakya et al., 2013). The significance of plant microbiomes and their role in stress tolerance has also been explored by several multi-omics techniques such as whole-genome and metagenomic analyses (Bai et al., 2015; Bulgarelli et al., 2015).

Several research studies lay emphasis on contemplating the use of plant growth-promoting (PGP) microorganisms to enhance the ability of host plants to sustain their productivity under stress conditions (Verma et al., 2015; Torbaghan et al., 2017; Albdaiwi et al., 2019; Ripa et al., 2019; Atieno et al., 2020; Rana et al., 2020). Several endophytic bacteria such as *Pseudomonas*, *Bacillus*, *Enterobacter*, *Burkholderia*, and *Azospirillum* colonize various plant species, and their promising benefits have been assessed over the years (Pieterse et al., 2012; Zipfel and Oldroyd, 2017; Afzal et al., 2019; Song et al., 2021; Suman et al., 2021; Zhang et al., 2021). Like other plant organs, the seed-harbored microbial endophytes have paramount potential, by establishing their existence in the host plant, generation after generation, through vertical transmission (Cope-Selby et al., 2017; Shade et al., 2017). The seed endophytes apparently foster seed germination and promote plant growth and development by utilizing various direct and indirect mechanisms, under several biotic and abiotic stress conditions (Santoyo et al., 2016; Shahzad et al., 2017; Rodríguez et al., 2018; Shearin et al., 2018).

Furthermore, PGP microbes are being considered an inexpensive alternative to resource-consuming agrochemicals in sustainable agriculture. Several kinds of commercial microbial formulations are available that specifically target nutrient mobilization, hormone production, and/or biocontrol of different pathogens (Berg et al., 2020). Such formulations are based on either a single or a consortium of microbial isolates and are not specific to crops. Hence, oftentimes, formulations showing excellent results under laboratory conditions fail to do so under field conditions. Wheat-associated microbial partnerships have been explored in several studies. Still, there are no evident reports on culturable and unculturable seed endophytic microbiomes of heat sensitivity variable wheat varieties. The epiphytic, endophytic, and rhizospheric bacterial diversity of wheat growing in six agroclimatic zones in India has been elucidated, and more than 200 diverse isolates were identified as PGP isolates (Verma et al., 2015; Suman et al., 2016; Verma et al., 2016; Verma and Suman, 2018; Sharma et al., 2022). Metagenomics of wheat rhizosphere has been studied for abiotic stress management and clearly indicated the future implications of beneficial bacteria in the rhizosphere to combat stress conditions (Ahlawat et al., 2018). The role of epiphytic pink-pigmented methylotrophic bacteria in wheat (*T. aestivum*) has been determined to enhance seed germination and seedling growth through the production of indole acetic acid (IAA) (Meena et al., 2012). Chakraborty et al. (2013) have shown the potential of osmotic stress-tolerant bacteria in water stress amelioration and plant growth promotion in wheat plants.

Seed microbiomes/endophytes are of particular interest as they are transmitted from generation to generation. By being seed-borne, these endophytes assure their presence in new plants (Truyens et al., 2015). Seed microbiomes have diverse interactions and are predicted to be an important biological resource for sustainable agriculture (Barret et al., 2015; Lugtenberg et al., 2016). In this study, the seed microbiomes of three wheat varieties having contrasting heat sensitivity indices (HSIs) were explored for culturable and unculturable microbial community composition and their probable beneficial functions, with the ultimate aim to identify beneficial microbiomes tolerant to high temperatures. Selected heat-tolerant PGP seed endophytes together with the unculturable microbiome would be formulated as small microbial communities to mitigate stress conditions in plants.

## 2 Materials and methods

### 2.1 Wheat seed sample collection

Seeds of three wheat varieties, GW322 (V1), HD3298 (V2), and HD3271 (V3) of HSI 1.26, 0.82, and 0.62, respectively, as per All India Coordinated Research Project (AICRP) reports on crop improvement (2016–2018), were collected. Among six wheat-growing regions of India, the variety V1 has been reported in the Central Zone and Peninsular Zone (PZ), and V2 and V3 have been reported in the North Western Plain Zone (NWPZ) and North Eastern Plain Zone (NEPZ) (**Table 1**). The seeds of selected wheat varieties were obtained through the Division of Genetics, ICAR–IARI, New Delhi, from different locations. The samples were taken in dry, clean, and sterile polythene bags and taken to the laboratory for further processing. The seed samples were collected randomly from five locations of their reported zones and pooled together to form a composite sample, which was used for further processing for the isolation of metagenome and culturable bacteria.

**Table 1:** General features of wheat varieties*.

Variety	Heat sensitivity/HSI	Temp. range (°C)	Sowing time	Duration (days)	Yield (t ha^–1^)	Indian Agro–climatic region	Locations for sampling	Latitude and longitude (coordinates)
GW 322	Heat^S^ 1.26	20–25	Nov 1–15, ES, TS	115 to 120	4.4	CZ and PZ	IARI RS, Indore (MP)	22°43′04″N 75°49′59″E
							UAS, Dharwad (Karnataka)	15°27’36’’N 75°00’37’’E
							IGKVV, Bilaspur (Chattisgarh)	22°04’43’’N 82°09’08’’E
							NIBSM, Raipur (Karnataka)	21°15’00’’N 81°37’47’’E
							ARS, Kota (Rajasthan)	25°12’49”N 75°51’53”E
HD 3298	Heat^T^ 0.82	25–30	Nov 25–Dec 5, LS, VLS	125 to 130	4.7	NEPZ and NWPZ	SVPUAT, Nagina (UP)	29°26’60”N 78°27’00”E
							ICAR–ATARI, Kanpur (UP)	26°26’59’’N 80°19’54’’E
							NDUAT, Faizabad (UP)	26°46’12’’N 82°09’00’’E
							RPCAU, Pusa (Bihar)	25°58’55”N 85°38’55”E
							IARI RS, Kalimpong (WB)	27°04’00’’N 88°28’00’’E
HD 3271	Heat^T^ 0.62	25–30	Jan 1–15, TS, LS, VLS	98 to 115	4.6	NEPZ and NWPZ	ICAR–ATARI, Kanpur (UP)	26°27′54″N 80°20′59″E
							RPCAU, Pusa (Bihar)	25°58’55”N 85°38’55”E
							GBPUAT, Pantnagar (UK)	29°03’00”N 79°31’00”E
							RARI, Durgapura (RJ)	23°54’00”N 89°01’00”E
							IARI RS, Kalimpong (WB)	27°04’00’’N 88°28’00’’E

*AICRP Crop Improvement Report (2016–2018), Heat^S^, Heat Susceptible; Heat^T^, Heat Tolerant; HSI, Heat Sensitivity Index; ES, Early sown; TS, Timely sown; LS, Late sown; VLS, Very late sown; CZ, Central Zone; PZ, Peninsular Zone; NWPZ, North Western Plain Zone; NEPZ, North Eastern Plain Zone.

### 2.2 Seed surface sterilization and processing for culturable and unculturable microbiome

#### 2.2.1 Surface sterilization of seed samples

One gram of wheat seeds from each of the composite samples of 3 varieties was taken in triplicate for the isolation of endophytic bacteria as well as for the isolation of genomic DNA. For surface sterilization, wheat seeds were first rinsed with sterile distilled water and washed with 70% ethanol for 30 s, followed by treatment with 1% sodium hypochlorite for 150 s. After that, a 70% ethanol wash was given again for 30 s and then the seeds were rinsed three to four times with sterile distilled water for the complete removal of traces of the sterilants used. Then, 100 µl of the last rinse water was plated on nutrient agar (NA) and incubated at 30 ± 2°C for 48–72 h to check the efficiency of sterilization.

#### 2.2.2 Culturable seed endophytic bacteria

The surface-sterilized seed samples of each wheat variety were immersed in 10 ml of sterile water for 1 h to soften them and then macerated well with 10 ml of sterile water using a sterilized pestle and mortar, yielding a 10^−1^ dilution. All the suspensions (10^−1^ dilutions) were then serially diluted at appropriate times in order to get a countable number of colonies. The suitable dilutions for each sample from all three varieties were spread plated on NA, TSA (tryptic soy agar), KBA (King’s B agar), and WMM (wheat matrix medium) developed previously in our laboratory (Sai Prasad et al., 2021). The plates were incubated at 30 ± 2°C for 4–5 days and observed each day for the appearance of bacterial colonies. The total number of colony-forming units (cfu) was counted for enumerating the population and all the distinct colonies emanating each day were selected as wheat seed endophytic bacterial (WSEB) isolate and purified on their respective media plates (Herrera et al., 2016).

#### 2.2.3 Seed metagenome and sequencing

The surface-sterilized seed samples were dried in a hot air oven at 50°C overnight to remove the moisture completely and then ground well with liquid nitrogen. One gram of powdered seed samples of each variety was weighed for the isolation of genomic DNA. The DNeasy^®^ Plant Mini Kit (Qiagen, the Netherlands) was used to extract the DNA according to the manufacturer’s protocol. After extraction, the DNA was tested for quality and purity. The quality was checked by using 0.8% agarose gel, and the purity was determined by a Nanodrop spectrophotometer (Thermo Scientific™). When the A260/280 ratio was 1.7–1.8, the pure DNA samples were sent for sequencing. The genomic DNA were sequenced by *de novo* whole-genome metagenome sequencing using the platform Illumina HiSeq X10 (150 bp × 2) through Agrigenome Labs Pvt. Ltd.

### 2.3 Phenotyping of wheat seed endophytic bacterial isolates

#### 2.3.1 Morphological and cultural characterization

The purified bacterial isolates were evaluated morphologically by observing their colony size, color, form, elevation, margin, texture, and opacity on TSA and WMM; the Gram and spore staining was based on Bergey’s Manual of Determinative Bacteriology (Holt et al., 1994). The distinctive isolates as selected morphotypes were transferred to NA media plates and then maintained on NA slants at 4°C for working stocks as well as in 30% glycerol stocks at −20°C for further use (Robinson et al., 2016).

The isolates were screened for growth in triplicate at different levels of pH (5.0, 7.0, and 9.0), salt (2.5%, 5%, 10%, and 15% of NaCl), and moisture stress at −0.05 MPa and −0.15 MPa (by using PEG 6000) with a main emphasis on temperature (30°C, 35°C, 40°C, 45°C, 50°C, and 55°C). For screening, each pure WSEB culture was inoculated in nutrient broth (NB) and incubated at 30 ± 2°C for 24 h. The freshly grown cultures (10 µl) were then spot inoculated on NA plates having different components for varying stress conditions and the plates were kept at 30 ± 2°C for incubation. In the case of temperature stress, the bacterial spotted plates were incubated at different temperatures as mentioned above.

#### 2.3.2 Functional characterization

Selected heat-tolerant WSEB isolates that were able to grow up to 55°C were evaluated for their potentiality to promote plant growth by screening them for nutrient solubilization/mineralization (P, K, and Zn) and production of siderophores and phytohormones. For qualitative screening, fresh broth cultures of WSEBs were prepared by inoculating the pure colonies in nutrient broth and incubating at 30 ± 2°C for 24 h to an approximate count of 10^6^ cfu ml^−1^. WSEB isolates were spotted on Pikovskaya’s medium plates amended with tricalcium phosphate [TCP; Ca_3_(PO_4_)_2_], as the unavailable form of P (Pikovskaya, 1948). The plates were incubated at 30 ± 2°C for 3–4 days. The appearance of a halo zone around the bacterial spot is taken as positive for P solubilization. Similarly, Aleksandrov agar medium containing potassium aluminosilicate (AlKO_6_Si_2_) (Hu et al., 2006) and Bunt and Rovira medium supplemented with 0.1% of zinc oxide (ZnO) and zinc phosphate [Zn_3_(PO_4_)_2_] (Bunt and Rovira, 1955) were used for testing K and Zn solubilization, respectively. Siderophore production was examined by observing the color change of the CAS (Chrom Azurol S) agar medium (Schwyn and Neilands, 1987). The production of IAA was observed by spot inoculating the WSEB isolates on Luria-Bertani (LB) agar plates supplemented with 100 µg ml^−1^ of L-tryptophan. After inoculation, each bacterial spot was covered with a small (1 cm) square piece of Whatman no. 1 filter paper and incubated at 37 ± 2°C for 5–7 days. After incubation, each filter paper piece was removed, dipped in Salkowski’s reagent (Gordon and Weber, 1951), and kept aside on another platform to observe the color change to pink and then to dark violet. All the assays mentioned were done in triplicate.

### 2.4 Genotyping and taxonomic analysis of cultured WSEB isolates

Selected heat-tolerant (heat^T^) WSEB isolates were subjected to molecular identification by sequencing the 16S rRNA gene. The genomic DNA of pure cultures of selected isolates was isolated by the Zymo Research (ZR) Bacterial DNA MiniPrep™ kit according to the manufacturer’s protocol (The Epigenetic Company). After extraction, the quality of isolated DNA was checked by agarose gel electrophoresis using 0.8% agarose. Furthermore, the 16S rRNA gene was amplified with the universal primers pA (27F) and pH (1492R) in a PCR thermocycler (peqSTAR). The reaction mixture contained the master mix (10 µl) with 10X Taq buffer, dNTPs (10 mM), MgCl_2_ (25 mM), Taq DNA Polymerase (1 U), forward and reverse primers (1.5 µl), nuclease-free water (5 µl), and bacterial genomic DNA (2 µl). The PCR conditions were set as follows: initial denaturation at 95°C for 2 min, followed by 35 cycles of denaturation at 95°C for 50 s, annealing at 53°C for 45 s, extension at 72°C for 90 s, and then final extension at 72°C for 7 min. Consequently, the PCR product was checked on agarose gel (1.2%) for confirmation of 16S rRNA gene amplification. The purified amplified products were sequenced by the Sanger dideoxy method (Agrigenome). The forward and reverse sequences obtained were assembled into contigs and checked for similarity with the identified bacterial database using NCBI-BLAST. Then, the database sequences with the maximum similarity were collected and aligned with the current sequences by ClustalW and the phylogenetic tree was constructed using the MEGAX software (Tamura et al., 2021).

### 2.5 Taxonomic analysis of uncultured microbiome

The raw data (forward and reverse sequences) of the metagenome sequences obtained in fastq format were uploaded to the MetaGenomics-Rapid Annotation using Subsystem Technology (MG-RAST) online web analysis server, and the details of the sequences submitted are given in **Supplementary Table 1**. The uploaded forward and reverse reads of each sample were initially joined together to obtain paired reads, and those paired reads were taken for further analysis. The nucleotide sequence data of the paired reads were then put through the MG-RAST pipeline with the following steps: preprocessing, dereplication, DRISEE, screening, gene calling, AA clustering 90%, protein identification, annotation mapping, and abundance profiling. The rRNA reads are identified and explored further through a separate flow in the pipeline by following steps such as rRNA detection, rRNA clustering 97%, and rRNA identification. The quality control steps include the removal of artificial duplicate reads, quality-based read trimming, and length-based read trimming. Those quality filtered reads were consequently submitted for annotation by the MG-RAST pipeline. After computation, the annotation results were collected and presented into the downstream pipelines through the analysis section where the annotations prepared as the abundance profiles were compared and community and metabolic reconstructions were carried out. The taxonomic annotations were compared with the standard RefSeq database, while the bacterial, fungal, and archaeal OTUs were separated using the filtering option. As the utmost aim was to determine the diversity and abundance of bacterial taxonomy, the filtered OTUs were analyzed further and the abundance profiles were created. Eventually, the results were displayed and exported in the desired format.

### 2.6 Statistical analysis

The statistical analyses of the culturable dataset were done using MS–EXCEL (version 2013). The phylogenetic tree was constructed using the neighbor-joining method on the MEGA11 software. The difference in overall microbial community composition of the three varieties was analyzed using the MG–RAST webserver. The results were represented by nonmetric MDS (NMDS) based on Bray–Curtis distances (Oksanen et al., 2017). Significance was accepted at an *α* level of 0.05 using Bonferroni correction. Venn diagrams and heatmaps for culturable and unculturable data were evaluated using R script. To distinguish the species richness, evenness, and dominance, the diversity indices were calculated at the genus level for metagenome and culturable data using the PAST software package v4.03 (Hammer et al., 2001). All the data presented are the mean of three replicates ± standard error (SE).

## 3 Results

### 3.1 Enumeration of endophytic bacteria from wheat seeds and selection of morphotypes

**Figure 1:**
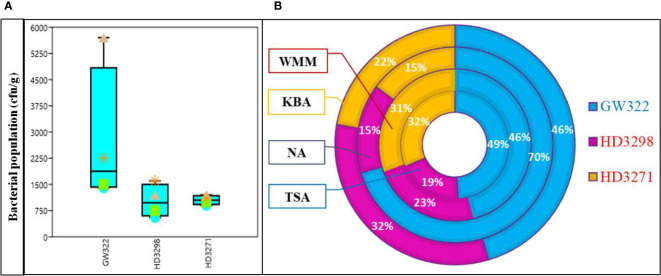
Culturable bacterial population from seeds of wheat varieties, heat^S^ (GW322) and heat^T^ (HD3298 and HD3271). **(A)** Box plot representing the population of bacteria (cfu g^–1^) in different media. *Triangle—NA, star—KBA, square—WMM, circle—TSA. **(B)** Percentage of the bacterial population in three varieties X growth media; NA, nutrient agar; KBA, King’s B agar; WMM, wheat matrix medium; TSA, tryptic soy agar. Values are the mean of three replications ± SE. Error bars denote the standard error.

Seeds of three wheat varieties with variable HSI were processed for the total bacterial population using different growth media. The total number of culturable bacteria among the three contrasting wheat varieties was found to be the highest in the heat^S^ variety (V1) followed by the heat^T^ variety V3 and by V2. Out of four different culture media evaluated, the population of bacteria was the highest in the NA, followed by KBA and then WMM, while TSA showed the least population for all three varieties (**Figure 1A**; **Supplementary Table 2**). Comparing the population among the heat^S^ and heat^T^ varieties, the highest number of bacteria was observed on NA plates of heat^S^ V1 (~70%, 57 × 10^2^ ± 148 cfu g^−1^), whereas in both heat^T^ varieties (V2 and V3), the population was relatively equal (~15% both) (**Figure 1B**). The second maximum population was determined by the KBA media, where V1 (46%, 22.5 × 10^2^ ± 89 cfu g^−1^) had the highest value followed subsequently by V2 (32%, 16 × 10^2^ ± 78 cfu g^−1^) and V3 (22%, 11 × 10^2^ ± 69 cfu g^−1^). In contrast, the number of bacterial colonies on TSA and WSA plates did not vary much and was comparatively higher in the case of WSA than TSA. Overall, the bacterial population was greater in the sequence V1 > V3 > V2 in both media (**Figure 1**). Morphometric analysis based on colony characteristics such as color, form, elevation, margin, size, appearance, texture, and elevation was carried out and the isolates that showed distinctly contrasting morphological characteristics were selected. A total of 44 WSEB, 19 from V1, 12 from V2, and 13 from V3, with varying morphotypes were taken for further screening.

### 3.2 Growth of wheat seed endophytic bacteria under different conditions

**Figure 2:**
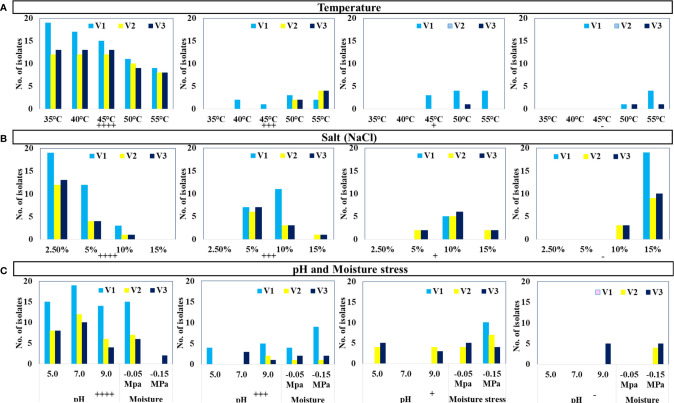
Growth of isolates (Nos.) under specified growth conditions: **(A)** temperature, **(B)** salt (NaCl), and **(C)** pH and moisture stress conditions (++++ Optimum growth, +++ Slightly less growth, + Minimum growth, - No growth).

The selected 44 distinct morphotypes of WSEB isolates from all three varieties were subjected to screening under different environmental conditions, viz., temperature (35°C to 55°C), pH (5.0 to 9.0), salt (up to 15%), and drought (up to 10%), with a main emphasis on heat tolerance. Surprisingly, all of the WSEB isolates from V2 and V3 were able to show their optimum growth up to 45°C (**Figure 2A**). Two isolates from V1 showed slightly lesser growth at 40°C, which was subsequently reduced at 45°C, at which one isolate showed a slightly reduced growth and three isolates showed minimum growth. Furthermore, three isolates from V1 and two each from V2 and V3 showed lesser growth at 50°C, while four isolates from V1 showed minimum growth. Yet, at 55°C, two isolates from V1, four from V2, and four from V3 showed lesser growth, while four from V1 showed minimal growth. Moreover, four isolates from V1 and one from V3 did not grow at 55°C (**Supplementary Table 3**). Hence, those isolates that showed optimum or slightly reduced growth, i.e., 11 isolates from V1 and 12 each from V2 and V3, were selected as heat^T^ WSEB isolates.

Selected WSEB isolates (44) could grow up to 5% of NaCl but only six isolates (three from V2 and three from V3) could grow even up to 15% NaCl (**Figure 2B**). Almost all the isolates were capable of ideal growth at pH of 5.0, 7.0, and 9.0, except some isolates showing moderate and minimum growth while five isolates from V3 did not grow at pH 9.0 (**Figure 2C**). In the case of drought, all the isolates were able to tolerate −0.05 MPa of moisture stress. Eventually, 35 WSEB isolates (S1 to S11 from V1, S12 to S23 from V2, and S24 to S35 from V3) were employed for further characterization of their PGP attributes.

### 3.3 Functional characterization of heat^T^ WSEB isolates for plant growth-promoting traits

**Figure 3:**
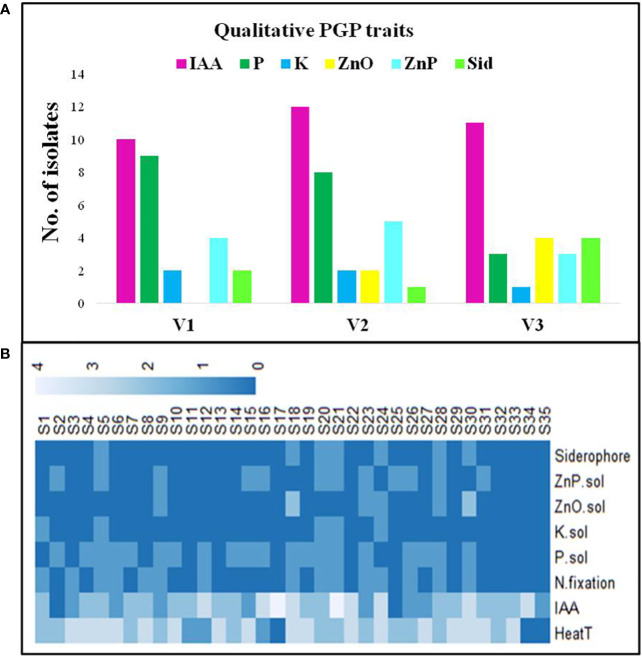
Plant growth-promoting (PGP) characteristics of WSEB isolates from three contrasting wheat varieties—heat^S^ (GW322) and heat^T^ (HD3298 and HD3271). **(A)** Graph representing the number of WSEB isolates showing PGP activities. **(B)** Bonitur scale of PGP attributes shown by selected heat^T^ WSEB isolates (IAA, indole acetic acid; N, nitrogen fixation; P, phosphorus solubilization; K, potassium solubilization; Sid, siderophore production; ZnO and ZnP—zinc oxide and zinc phosphate solubilization).

Selected WSEB isolates were screened qualitatively for different PGP traits. All the selected isolates except S2 and S25 produced IAA in the low (S20, S21, and S35), medium (S1, S11, S13, S14, S23, S28, S29, and S32) and high range (**Supplementary Table 4**; **Figure 3A**). Out of the selected isolates, 10 from V1, 12 from V2, and 11 from V3 showed IAA production (**Supplementary Table 5**). On the other hand, the P solubilization was exhibited by nine isolates from V1, and eight and three isolates from V2 and V3, respectively. Only five isolates (S5, S11, S12, S13, and S4), two each from V1 and V2 and one from V3, had K solubilization potential, while in the case of Zn solubilization, zinc oxide was solubilized by 6 isolates (S19, S23, S24, S27, S28, and S30) and zinc phosphate was solubilized by 12 isolates (4, 5, and 3 from V1, V2, and V3, correspondingly). In the case of siderophore production, two isolates (S5 and S11) from V1, S12 from V2, and S24, S27, S28, and S30 from V3 showed a positive reaction. By considering the level of qualitative PGP traits exhibited by each isolate, a bonitur scale was prepared (**Figure 3B**) and the isolates were ranked based on their maximum PGP potential.

### 3.4 Taxonomic diversity of cultured heat^T^ WSEB isolates

**Figure 4:**
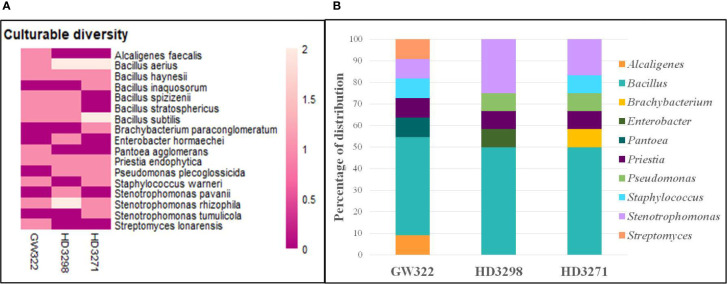
Abundance and diversity of WSEB isolates from wheat varieties—heat^S^ (GW322) and heat^T^ (HD3298 and HD3271). **(A)** Heatmap representing the abundance of culturable bacterial diversity (species level). **(B)** Relative genera abundance (%) of identified WSEB isolates.

**Figure 5:**
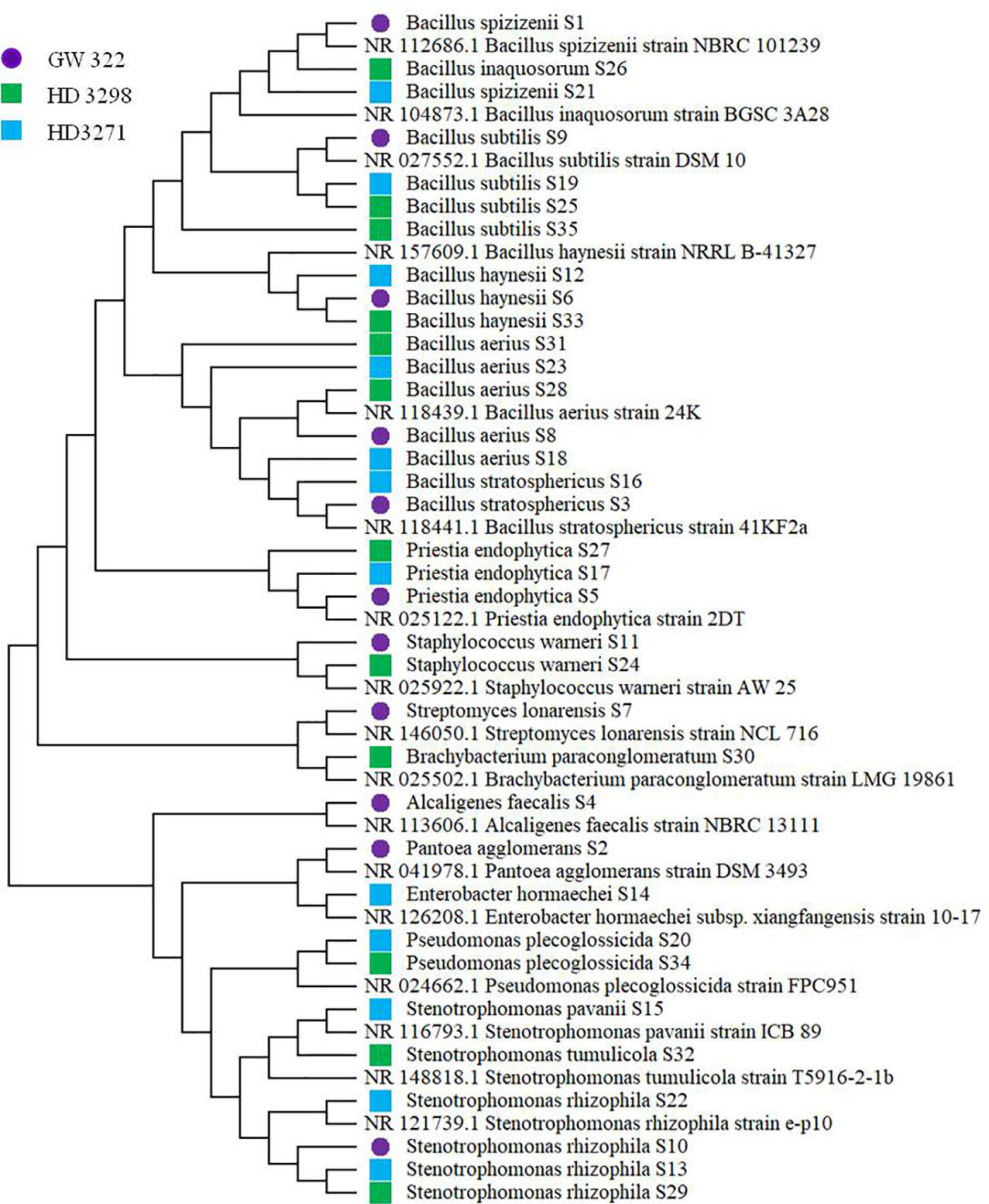
Phylogenetic tree of identified heat^T^ WSEB isolates from wheat varieties—heat^S^ (GW322) and heat^T^ (HD3298 and HD3271) [constructed using neighbor joining algorithm with 1,000 bootstrap values. *Purple filled circle (S1 to S11)—isolates from heat^S^ (GW322) variety, green filled square (S12 to S23)—isolates from heat^T^ (HD3298) variety, and blue filled square (S24 to S35)—isolates from heat^T^ (HD3271) variety].

To ascertain the diversity of culturable bacterial flora, the contigs of the 16S rRNA gene of all selected WSEB isolates were subjected to BLAST analysis, which revealed that all the sequences have >97% similarity with the sequences available in the GenBank database and belonged to 10 different genera, viz., *Alcaligenes*, *Bacillus* (six species) *Brachybacterium*, *Enterobacter*, *Pantoea*, *Priestia*, *Pseudomonas*, *Staphylococcus*, *Stenotrophomonas* (3 species), and *Streptomyces* (**Figure 4A**; **Supplementary Table 6**), of the classes Betaproteobacteria, Gammaproteobacteria, Bacilli, and Actinomycetia. The phylogenetic analysis revealed that the isolates S10 of V1, S13 and S22 of V2, and S29 of V3 were identified as *Stenotrophomonas rhizophila* (**Figure 5**). *Pantoea agglomerans* was found with 98.13% similarity for S2, which was present only in V1. In the Bacilli group, *Bacillus inaquosorum* (S26), *Bacillus spizizenii* (S1 and S21), and *Bacillus stratosphericus* (S3 and S16) were also detected in V1 and V2, whereas *Bacillus subtilis* (S9, S19, S25, and S35), *Bacillus hayneii* (S6, S12, and S33), and *Bacillus aerius* (S8, S18, S23, S28, and S31) were observed in all three varieties. The isolates S5, S17, and S27 were matched with *Priestia endophytica* common in all varieties (**Supplementary Table 6**). Other than the *Bacillus* spp., S11 and S24 were identified in other Firmicutes and *Staphylococcus warneri* and spotted only in heat^T^ varieties. In the case of *Stenotrophomonas*, *S. pavanii* was peculiar to V2 and *S. tumulicola* was present only in V3. However, isolate S30 was complementary to *Brachybacterium paraconglomeratum* with 99.72% similarity, which was specific to the heat^T^ V3. Likewise, another Actinobacteria, *Streptomyces lonarensis* (S7), was specific to V1. Exceptionally, most of the chosen isolates (24) were Gram-positive and *Bacillus* was the predominant genus covering approximately 50% of the culturable diversity in all three varieties (**Figure 4B**). The 16S rRNA gene sequences of all the identified isolates were submitted to the NCBI database under accession numbers OP782593–OP782627.

### 3.5 Taxonomic diversity of unculturable seed metagenome

The whole-genome shotgun sequencing of the metagenome yielded a total of approximately 10,154,754 ± 685,263 sequences as an average in all three samples (229 ± 3 bp in length), which contained an average of 106,296 ± 9,612 sequences with rRNA genes (**Table 2**). When those sequences were subjected to MG–RAST and subsequent analysis, it was found to have a total of 625 operational taxonomic units (OTUs) belonging to bacteria (523 OTUs), archaea (41 OTUs), and fungi (61 OTUs), while matching with the RefSeq database.

**Table 2:** Statistics of metagenome sequences for MG–RAST analysis platform.

Summary statistics of submitted sequences	Seeds of Wheat Varieties		
	GW322 Heat^S^	HD3298 Heat^T^	HD3271 Heat^T^
Total bp Count	2,562,025,112 bp	2,317,493,646 bp	2,097,209,168 bp
Sequences Count	11,414,000	9,993,617	9,056,645
Mean Sequence Length	224 ± 43 bp	232 ± 40 bp	232 ± 41 bp
Mean GC percent	47 ± 9%	47 ± 9%	47 ± 9%
Artificial Duplicate Reads: Sequence Count	1,953,345	1,264,385	1,124,474
Post QC: bp Count	2,106,281,345 bp	2,007,313,383 bp	1,819,629,751 bp
Post QC: Sequences Count	9,337,341	8,620,077	7,828,322
Post QC: Mean Sequence Length	226 ± 43 bp	233 ± 40 bp	232 ± 41 bp
Post QC: Mean GC Percent	47 ± 9%	48 ± 9%	48 ± 9%
Processed: Predicted Protein Features	5,883,627	5,568,431	5,085,028
Processed: Predicted rRNA Features	53,286	41,578	40,993
Alignment: Identified Protein Features	656,621	637,422	574,678
Alignment: Identified rRNA Features	3,795	2,488	2,054
Annotation: Identified Functional Categories	Undefined	Undefined	Undefined
Total DRISEE error	4.137%	4.162%	5.426%

The microbial composition of all three contrasting wheat varieties revealed that the number of fungal OTUs was highly abundant, followed by bacteria and then archaea. In the heat^S^ variety, V1 had the highest number of OTUs, of which Fungi were the dominant group. Bacteria were the second dominant domain and Archaea were observed to be the least abundant (**Table 3**). In contrast, Bacteria were most abundant in both heat^T^ varieties and Fungi were the second most, followed by Archaea. Yet, the number of bacterial OTUs was higher in the order of V2 > V1 > V3.

**Table 3:** Taxonomy abundance of microbial OTUs in the seed metagenome.

Microbial		Seeds of Wheat Varieties		
Domain	Phyla	GW322 Heat^S^	HD3298 Heat^T^	HD3271 Heat^T^
Archaea		286	221	293
Bacteria		17,281	20,692	16,849
	Actinobacteria	3,142 (18.18%)	2,563 (12.39%)	3,096 (18.38%)
	Firmicutes	4,053 (23.45%)	3,602 (17.41%)	2,986 (17.72%)
	Proteobacteria	9,426 (54.55%)	13,856 (66.96%)	10,093 (59.90%)
	Others	660 (3.82%)	671 (3.25%)	674 (4.00%)
Eukaryota		26,230	8,389	8,611

The bacterial OTUs were separated from the dataset using RefSeq comparison, where a total of 25 different phyla were unveiled, and it was found that all three varieties were dominated by the phyla Proteobacteria containing more than 50% of OTUs (**Supplementary Figure 1**). The heat^T^ V2 had the highest number of OTUs (66.96%) and the heat^S^ V1 had the lowest (54.44%). Next to Proteobacteria, Firmicutes were the second dominant group of bacteria found among the three varieties, where V1 contained the highest abundance (23.45%) and V2 and V3 had almost a similar rate of abundance (~17.5%). Actinobacteria were observed to be the third most abundant phyla with approximately 12% of bacterial OTUs in V2 as well as approximately 18% in V1 and V3. Seventy-five percent of OTU abundance was shared by the phyla Proteobacteria, Firmicutes, and Actinobacteria, while Cyanobacteria and Bacteroidetes had more than 100 OTUs per sample (**Table 4**). Specifically, the other phylum, Fusobacteria, was highly abundant in heat^T^ V3 with 104 OTUs, but the other varieties V2 and V1 had only 9 and 2 OTUs, respectively. Another distinct aspect observed was the presence of phyla Elusimicrobia, Fibrobacteres, and Synergistetes only in heat^T^ varieties. In the Proteobacteria group, Gammaproteobacteria were profoundly abundant and the other classes Betaproteobacteria, Alphaproteobacteria, and Deltaproteobacteria also had significant abundance. Another group consisting of the class Actinobacteria was prominent as well (**Supplementary Figure 2**). Moreover, the Firmicutes containing Bacilli and Clostridia were also prevalent in all three varieties and abundant in the heat^S^ variety.

**Table 4:** Phyla-wise distribution of bacterial abundance among three varieties.

Microbial		Seeds of Wheat Varieties		
Domain	Phyla	GW322 Heat^S^	HD3298 Heat^T^	HD3271 Heat^T^
Bacteria	Proteobacteria	9,426	13,856	10,093
Bacteria	Firmicutes	4,053	3,602	2,986
Bacteria	Actinobacteria	3,142	2,563	3,096
Bacteria	Cyanobacteria	305	296	251
Bacteria	Bacteroidetes	100	139	119
Bacteria	Chloroflexi	83	68	54
Bacteria	Planctomycetes	52	38	52
Bacteria	Verrucomicrobia	23	12	12
Bacteria	Spirochaetes	18	28	10
Bacteria	Acidobacteria	16	26	18
Bacteria	Chlorobi	11	13	11
Bacteria	Deinococcus-Thermus	10	10	8
Bacteria	Fusobacteria	9	2	104
Bacteria	Thermotogae	6	12	7
Bacteria	Tenericutes	5	4	1
Bacteria	Deferribacteres	4	2	2
Bacteria	Chlamydiae	3	2	4
Bacteria	Chrysiogenetes	3	1	3
Bacteria	Gemmatimonadetes	3	2	3
Bacteria	Nitrospirae	3	7	3
Bacteria	Aquificae	2	2	5
Bacteria	Lentisphaerae	2	1	2
Bacteria	Elusimicrobia	0	1	0
Bacteria	Fibrobacteres	0	0	2
Bacteria	Synergistetes	0	1	3

### 3.6 Variety-wise distribution of bacterial taxonomy at the genus level

Considering the genera-level abundance, *Clostridium* of Firmicutes was the most abundant bacteria found in heat^S^ V1 (13.16% of bacterial OTUs) (**Supplementary Figure 3A**), whereas *Stenotrophomonas* (Gammaproteobacteria) was dominant in both heat^T^ varieties (13.07% in V2 and 11.73% in V3). *Enterobacter* was detected as the third most dominant in all the varieties (7.10%–7.99% of bacterial OTUs), which was further followed by the *Bacillus* group (4.03%–6.22% of bacterial OTUs) and then by the Actinobacteria, *Streptomyces*, and *Bifidobacterium* (**Supplementary Figure 3B**). In the case of heat^S^ V1, 33.20% of Actinobacteria belonged to *Streptomyces*, whereas V2 had 30.59% and V3 had 26.68%. In the heat^T^ V3, 32.66% of Actinobacteria belonged to *Rhodococcus*, while both *Bifidobacterium* and *Rhodococcus* shared ~23.5% each of Actinobacteria in V1. Together, *Streptomyces*, *Bifidobacterium*, *Rhodococcus*, and *Mycobacterium* consisted of more than 90% of Actinobacteria in V1 and V3, and approximately 84% in V2. In contrast, *Clostridium* was found to be 63.83% of Firmicutes in V2, and approximately 56% in V1 and V3. The next dominant genus, *Bacillus*, was highly abundant in V3, followed by V1 and V2, covering 28.20%, 26.52%, and 23.15% of Firmicutes, respectively. Furthermore, approximately 19.5% of Proteobacteria in heat^T^ V2 and V3 consisted of *Stenotrophomonas*, but was approximately 15.9% in heat^S^ V1 (**Supplementary Figure 3C**). In comparison, 13.7% of the Proteobacteria were substantiated by *Enterobacter* in V1, whereas it was approximately 11.9% in V2 and V3. Almost 75% of the Proteobacteria in all three varieties were substituted by 20 different genera from Gammaproteobacteria (*Stenotrophomonas*, *Enterobacter*, *Xanthomonas*, *Pseudomonas*, *Magnetococcus*, *Beggiatoa*, *Acinetobacter*, *Escherichia*, *Citrobacter*, *Vibrio*, *Klebsiella*, *Salmonella*, and *Pantoea*), Betaproteobacteria (*Achromobacter*, *Burkholderia*, *Ralstonia*, *Bordetella*, and *Delftia*), and Alphaproteobacteria (*Roseobacter* and *Brevundimonas*).

**Figure 6:**
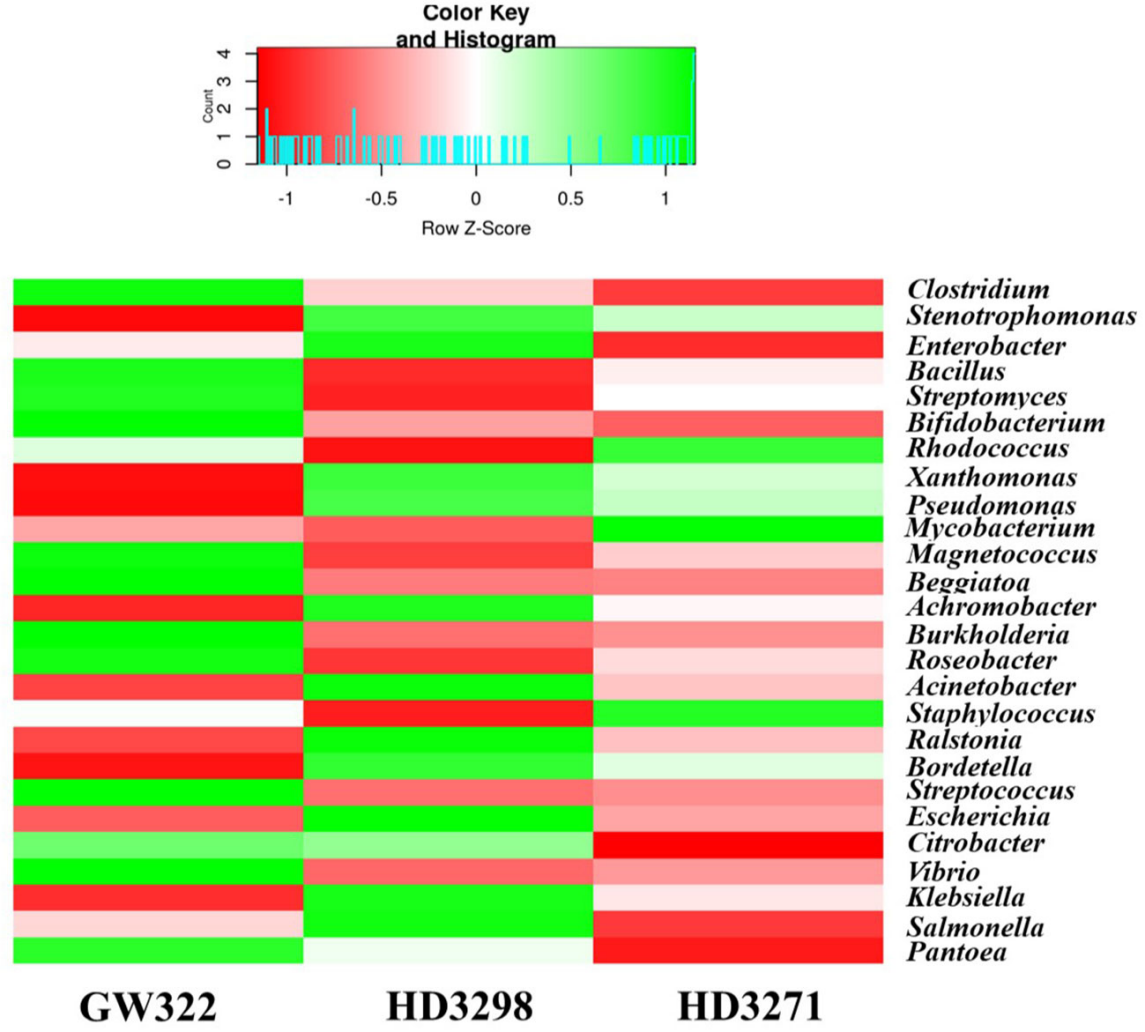
Heatmap showing the abundance profile of dominant bacteria (top 25 genera) from the metagenomic analysis.

Moreover, the number of OTUs of some bacteria like *Streptococcus*, *Pantoea*, and *Cellulosilyticum* was much higher in the heat^S^ than in the heat^T^ variety and the abundance profile of the most dominant genera is presented in **Figure 6**. There were several bacteria with a lesser number of OTUs, and some of them are specific only to the heat^S^ and heat^T^ varieties. A total of 25 different genera, including *Ktedonobacter*, *Heliobacterium*, *Xylanimonas*, *Desulfotalea*, *Moritella*, *Bermanella*, *Kosmotoga*, *Parascardovia*, *Kordia*, and *Xenorhabdus*, were limited only to the heat^S^ variety, while 36 genera, including *Brevibacterium*, *Nakamurella*, *Thermomicrobium*, *Acetivibrio*, *Azospirillum*, *Methylophaga*, *Spirochaeta*, *Ureaplasma*, *Fervidobacterium*, and *Thermotoga*, were peculiar to both the heat^T^ varieties. The top 25 genera (**Figure 6**) (including all bacterial phyla) of all three varieties have covered more than 75% of the abundance.

### 3.7 Diversity indices of bacterial community composition

**Figure 7:**
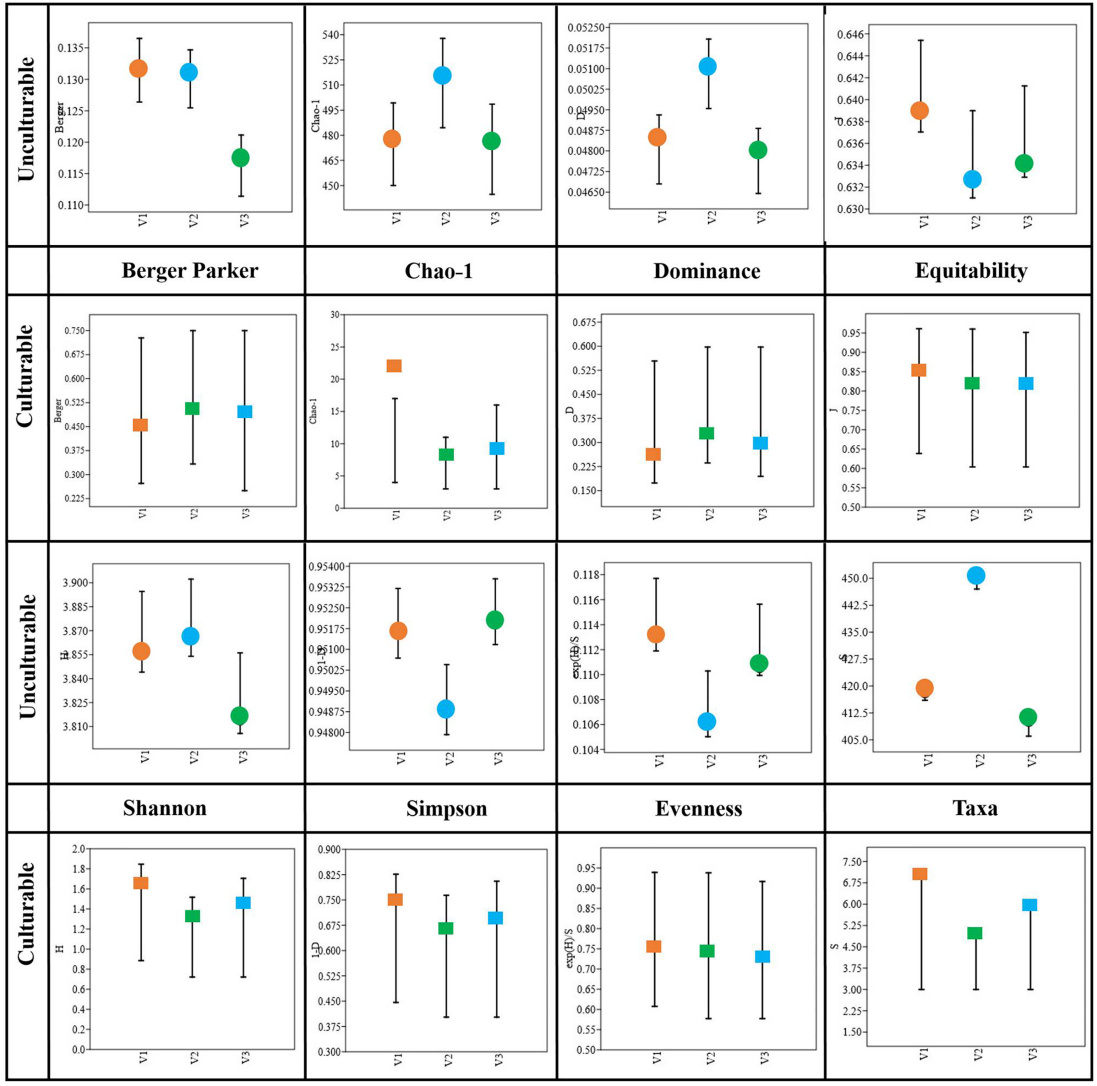
Diversity indices of bacterial taxonomy in heat^S^ (GW322) and heat^T^ (HD3298 and HD3271) wheat varieties.

The diversity of both culturable and unculturable bacterial communities was calculated using the measures of Shannon (H), Simpson (1-D), Dominance (D), Evenness (e^H^/S), and species richness (S_chao-1_) indices, and the results are presented in **Figure 7**. With respect to the unculturable community composition, the Taxa (S) abundance was higher (S = 450) in V2, and so is the dominance index (D = 0.05), while there was no significant difference between the dominance of V1 and V3. The Simpson index is directed against the evenness of the communities, in which the heat^S^ V1 and heat^T^ V3 had higher values and V2 had comparatively lower values. However, the H-index is represented in relation to the richness of the communities, where V2 had the highest index followed by V1 and V3 (**Supplementary Table 7**). Similarly, the S_chao-1_, representing the overall predicted microbial richness, was high (514.7) for V2, whereas not much significant difference was observed between V1 and V3. On the other hand, while considering the culturable diversity, the taxa abundance (S = 7) of V1 was higher than V3 (S = 6) followed by V2 (S = 5). The dominance index (D) was much lesser in heat^S^ V1 when compared to heat^T^ varieties. Furthermore, the bacterial communities exhibit high evenness in V1 (e^H^/S = 0.76) compared to V2 (0.74) and V3 (0.73) under culturable conditions, where it is lowest in V2 (0.106) followed by V3 (0.111) and V1 (0.113) under unculturable conditions. Nevertheless, the species richness (S_chao-1_) was higher in the order V1 > V3 > V2. All the diversity indices of culturable communities were based on the isolates selected and may be biased by the isolation and selection procedures.

### 3.8 Similarities and differences of the culturable and unculturable microbiomes

**Figure 8:**
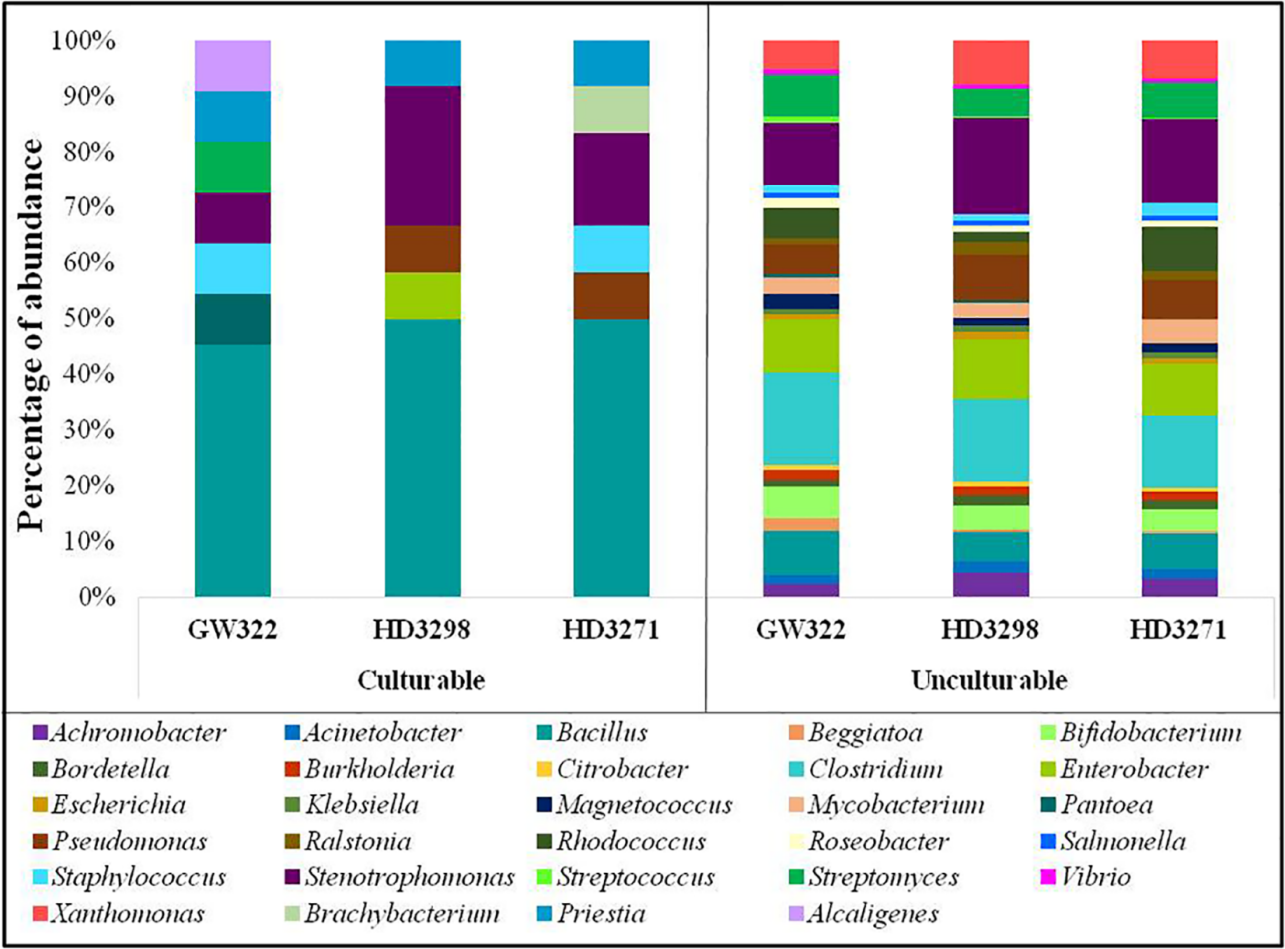
Comparative abundance of bacteria in culturable and unculturable communities of heat^S^ (GW322) and heat^T^ (HD3298 and HD3271) wheat varieties.

The bacterial diversity in the culturable and unculturable microbiomes has shown that *Stenotrophomonas* was highly abundant in the metagenome (8.70%, 13.08%, and 11.73% of V1, V2, and V3, respectively), but its proportion was reduced upon culturing to 9.09%, 25%, and 16.67% in V1, V2, and V3, respectively. Still, the abundance of bacteria was the highest in the order of V2 > V3 > V1, in both culturable and unculturable communities (**Figure 8**). As the isolation procedures focused mainly only on culturing the aerobic bacteria, *Clostridium*, which was predominant in the metagenome, was eliminated in the culturable community. In addition, *Bacillus* spp. as fastidious Gram-positive spore formers were able to dominate the culturable communities with almost 50% of habitation in the tested varieties. Still, the heat^T^ V2 and V3 had higher dominance for *Bacillus* than the heat^S^, but it was found to be in contrast to the metagenome, as more dominance for *Bacillus* was observed in heat^S^ V1 (**Figure 8**). *Streptomyces* recovered only in V1 in the case of culturable communities, though in unculturable communities, it shared 6.04%, 3.79%, and 4.90% in V1, V2, and V3, respectively. In contrast, *Pseudomonas* was present only in the heat^T^ varieties in the culturable communities and *Pantoea* was identified only in the heat^S^ variety in the culturable communities, whereas its presence was noted in the metagenome of all three varieties. Distinctly, *Alcaligenes* was not identified from the metagenome but was found in the heat^S^ V1 in cultured bacteria, while *Brachybacterium* was cultured in V3 but observed in the metagenome of V1 and V2 with high dominance in V2. The genus *Priestia* was not noticed in the metagenome of any variety, but constituted 9.09% in V1 and 8.33% each in V2 and V3. In place of 9.09% and 8.33% each for V1, V2, and V3, respectively. Similarly, the genus *Enterobacter* was present only in V2 in the isolated cultures, while it was relatively dominant in the unculturable communities, contributing up to 7.48%, 7.99%, and 7.10% in V1, V2, and V3, correspondingly. On the other hand, *Staphylococcus* was detected only in V1 and V3, whereas the metagenome of all three varieties was present, with comparably lesser dominance in V2. Altogether, culturable bacteria abundance was substantially similar to the unculturable abundance of the same genera.

## 4 Discussion

Endophytes are the key class of plant symbionts subsisting inside the cells, such as roots (rhizosphere), leaves (phylloplane), stems (laimosphere and caulosphere), fruits (carposphere), seeds (spermosphere), and flowers (anthosphere) (Clay and Holah, 1999; Lindow and Brandl, 2003; Saikkonen et al., 2004; Shahzad et al., 2017), without causing any diseases (Brader et al., 2017), and are bonded together throughout their life cycle. The diversity of endophytic bacteria in the seeds of wheat varieties having contrastive heat sensitivity was assessed by culturable and unculturable approaches in this study. The culturable diversity showed that the heat^S^ variety held a larger population of bacteria compared to the heat^T^ variety, which might be due to the selection pressure offered by the different genotypes over the bacterial communities. Moreover, the susceptible genotypes probably require assistance to sustain their life, which diverse groups of microorganisms could provide either by enhancing the plant nutrient acquisition and biocontrol or by improving the genetic makeup of the plants through certain stress-responsive genes and molecular chaperones. Yet, there is no proof confirming this cause until now. However, it was stated that seed endophytes have a wide extent of colonization and could be found either in lesser or greater numbers (Mundt and Hinkle, 1976; Rijavec et al., 2007). Also, epiphytic, endophytic, and rhizospheric bacterial diversity of wheat growing in six agroclimatic zones in India has been determined by various studies (Verma et al., 2013; Verma et al., 2014; Verma et al., 2015; Suman et al., 2016; Verma et al., 2016; Verma and Suman, 2018) and more than 200 diverse isolates were identified as PGP isolates. In this study, the endophytic bacteria from three contrasting wheat varieties were isolated using four different growth media, whereas the NA media held the highest population ranging from 12×10^2^ to 57×10^2^ cfu g^−1^ of seeds. This was in contrast with the observations by Robinson et al. (2016), where the serially diluted samples of surface-sterilized seeds rendered no bacterial colonies. Furthermore, in rice seeds, the population of bacteria was detected up to 3.5 × 10^5^ cfu g^−1^ of fresh tissue (Hardoim et al., 2012), and the aerial tissues had been expeditiously inhabited by the seed-borne endophytes. Likewise, Sai Prasad et al. (2021) and Manias et al. (2020) reported that it would be sensible to use different growth media for the isolation of endophytes, especially for analyzing abundance and diversity.

Thereafter, the bacterial colonies showing different morphological characteristics were purified and subjected to cultural characterization to evaluate their capability to grow at a wider range of environmental conditions in this study, while several abiotic stress factors ranging from high temperature, drought, and salinity to oxidative stress and heavy metal toxicity are inhibitory to the plants’ growth. The ability of osmotic stress-tolerant bacteria to promote plant growth and ameliorate the water stress in wheat has been deciphered by Chakraborty et al. (2013). However, it was demonstrated that most of the WSEB isolates from three different varieties were able to grow under diverse circumstances. The WSEB isolates from heat^T^ varieties were well established under an increasing temperature range when compared to the heat^S^ variety. The ability of these endophytes might increase the bounds of temperature tolerance of heat^T^ varieties (Dastogeer et al., 2022). This was in line with the studies that growth of spot inoculated purified microbial isolates on medium plates under selection pressure of some chemicals or incubation conditions is considered as tolerant to respective stress condition. (Zhang et al., 2006; Fan et al., 2018).

PGP endophytic microorganisms help regulate the growth of plants through the production of various phytohormones such as auxins, gibberellins, and cytokinins (Santoyo et al., 2016). The production of IAA by the epiphytic pink-pigmented methylotrophic bacteria in wheat has been proven to enhance seed germination and seedling growth (Meena et al., 2012). Studies have reviewed that auxin, as an effector molecule, regulates the interaction between bacteria and plants, as well as between bacteria (Lambrecht et al., 2000; Spaepen and Vanderleyden, 2011). The endophytes also aid in solubilizing the unavailable form of nutrients such as phosphorus, potassium, iron, and zinc and make them available to plants by facilitating absorption. Moreover, the siderophore produced by the endophytic microbes benefits the plants through both direct and indirect mechanisms, i.e., by promoting iron acquisition as well as by increasing the competition for available iron and defending against pathogenic organisms (Bouizgarne, 2013). However, most of the heat^T^ WSEB endophytes isolated from heat^T^ and heat^S^ varieties had the tendency to produce more IAA and solubilize phosphorus, while some were able to grow in an N-free medium and solubilize zinc phosphate. Relatively few WSEB isolates could solubilize potassium and zinc oxide and produce siderophores.

**Figure 9:**
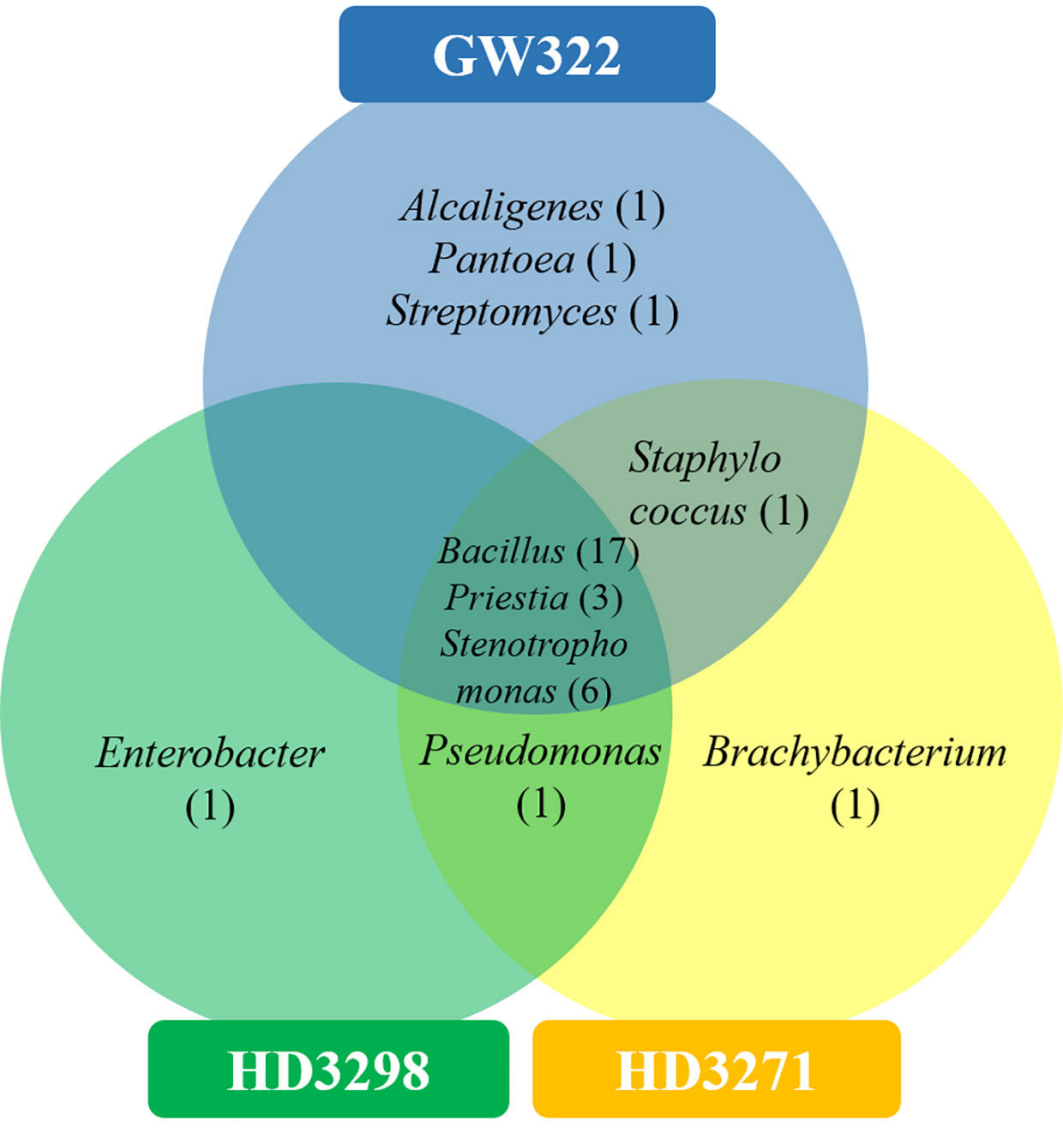
Venn diagram showing the genus-level distribution of culturable WSEB isolates from heat^S^ (GW322) and heat^T^ (HD3298 and HD3271) wheat varieties.

Subsequently, the 16S rRNA gene of all heat^T^ WSEB isolates was amplified and identified in a total of 10 different genera, namely, *Alcaligenes*, *Bacillus*, *Brachybacterium*, *Enterobacter*, *Pantoea*, *Priestia*, *Pseudomonas*, *Staphylococcus*, *Stenotrophomonas*, and *Streptomyces*, with more diversity in heat^S^ V1 (**Figure 9**). Three genera, viz., *Bacillus*, *Priestia*, and *Stenotrophomonas*, were common in all three varieties. Some genera were specific to particular varieties, such as *Alcaligenes*, *Pantoea*, and *Streptomyces* specific to V1, *Enterobacter* specific to V2, and *Brachybacterium* specific to V3. Then, the phylogenetic tree was constructed, having a 1,000 bootstrap value by the neighbor-joining method. However, similar studies have been conducted in which phylogenetic trees and distance matrices were made by maximum likelihood algorithms using the MEGA software (Thomas, 2011; Fan et al., 2018; Shan et al., 2018; Sánchez-Cruz et al., 2019). The bacterial communities extensively found as seed endophytes belonged to the phylum Proteobacteria. Other phyla like Actinobacteria, Firmicutes, and Bacteroidetes were encountered to a lesser degree (Hardoim et al., 2015). The most commonly reported genera from the plant seeds include *Bacillus* and *Pseudomonas*, while *Paenibacillus*, *Micrococcus*, *Staphylococcus*, *Pantoea*, and *Acinetobacter* have also been reported (Truyens et al., 2015; Herrera et al., 2016). Large amounts of endospore-forming Gram-positive bacteria are capable of surviving inside the seeds for a longer period (Luna et al., 2010). Likewise, in this study, *Bacillus* and *Priestia* (derived from *Bacillus*) together contributed to more than 50% of the identified WSEB isolates, which might be attributed to the fastidious nature of their growth and the spore-forming ability to survive.

In general, barely a limited number of potential bacteria are allowed to enter and colonize inside parts of the plant, which confer the specificity of endophytic rather than rhizospheric microbiome. The endophytic microbiome advance their connection mainly during seed germination and early development (Johnston-Monje et al., 2014), making an integrated network in the plant interior, which remains persistent through all the phases of the plant growth cycle (Podolich et al., 2015). Unraveling the probable beneficial functions of the plant microbiome by high-throughput sequencing approaches together with the culture-dependent approaches would pave the way towards understanding their interactions that could be labored to improve plant growth and health (Mendes et al., 2013; Suman et al., 2021). The composition of seed microbiome from drought-tolerant and drought-susceptible wheat lines was evaluated under rainfed and drought conditions by using culture-dependent and metagenomic methods (Hone et al., 2021). The assembly of bacterial and fungal microbiota in domesticated and wild wheat species was regulated by the selection factors such as plant habitat and host genetics (Bulgarelli et al., 2012; Edwards et al., 2015). These differences showed greater degrees of variance in the community structure (Özkurt et al., 2020). Likewise, our study has clearly depicted that the fungal OTUs were significantly higher than the bacterial OTUs, and also the bacterial communities differed according to the hosts’ ability to tolerate heat stress, and this is not very surprising as the stress tolerance pressure imparts a higher selective force on the community assembly (Peiffer et al., 2013; Mendes et al., 2014; Ofek-Lalzar et al., 2014; Coleman-Derr et al., 2016).

The evolution of bacterial communities in rhizospheric samples from wheat fields was studied by Donn et al. (2015). Ahlawat et al. (2018) deduced the eventual significance of rhizospheric bacteria to combat stress conditions by studying the metagenomics of wheat rhizosphere. Germida and Siciliano (2001) have shown that the taxonomic diversity of bacteria congregated to the roots of modern, recent, and ancient cultivars of wheat. A total of 30 different OTUs that belonged to Alphaproteobacteria, Betaproteobacteria, Deltaproteobacteria, Gammaproteobateria, Actinobacteria, Bacilli, Clostridia, and uncultivable bacteria were identified from the wheat rhizosphere (Velázquez-Sepúlveda et al., 2012). Similarly, this investigation revealed the distribution of bacterial OTUs in three dominant phyla, viz., Proteobacteria, Actinobacteria, and Firmicutes, along with minimum contribution from several other phyla such as Acidobacteria, Aquificae, Bacteroidetes, Chlorobi, Chloroflexi, Cyanobacteria, Chrysiogenetes, Deferribacteres, Deinococcus-Thermus, Elusimicrobia, Fibrobacteres, Fusobacteria, Gemmatimonadetes, Lentisphaerae, Nitrospirae, Planctomycetes, Spirochaetes, Synergistetes, Tenericutes, Thermotogae, and Verrucomicrobia.

Considering the class-level distribution, the most abundant class was Gammaproteobacteria, followed by Alphaproteobacteria (Hardoim et al., 2015; Rahman et al., 2018) and Bacilli (Comby et al., 2016). It was also noted from studies on barley, rice, bean, and maize endosperms that Proteobacteria, Actinobacteria, and Firmicutes were usually found to be dominant (Kaga et al., 2009; Ruiza et al., 2011). This was in agreement with the current study in which the Gammaproteobacteria had the highest number of OTUs, while contrasting evidence showed the distinct dominance of Actinobacteria and Clostridia as the second and third highest, respectively, followed by Bacilli, Betaproteobacteria, and Alphaproteobacteria. The unclassified classes derived from phyla Proteobacteria and Cyanobacteria also offered approximately 250 OTUs per sample, whereas Deltaproteobacteria came next to the unclassified group.

When comparing the presence of Zn-mobilizing species such as *Adhaeribacter*, *Janthinobacterium*, *Massilia*, and *Pseudomonas* in rhizospheric and bulk soil of wheat, it was observed that the relative abundance was higher in the rhizosphere that could articulate the local community structure to improve the plant growth by mobilizing nutrients (Wang et al., 2021). *Acinetobacter* was highly prominent, while *Pantoea*, *Pseudomonas*, and *Paracoccus* were also detected from the endosperm of *T. aestivum* cv. “Hondia”. *Acinetobacter*, *Micrococcus*, and *Staphylococcus* were also viewed by the endosperm as endophytes (Truyens et al., 2015; Soldan et al., 2019). Distinctively, in our study, *Clostridium* was obtained as the predominant genus from heat^S^ V1, while *Stenotrophomonas* was predominant in heat^T^ V2 and V3, followed by *Enterobacter*, *Bacillus*, and *Streptomyces*. Moreover, the variation in relative abundance and specificity of bacterial communities was also found between the tested heat^T^ and heat^S^ varieties, where a total of 36 different genera were specific only to the tolerant variety as well as 25 different genera specific to the susceptible variety. An interesting feature with this specificity was that the unique genera (*Thermomicrobium*, *Acetivibrio*, *Methylophaga*, *Fervidobacterium*, and *Thermotoga*) found in heat^T^ varieties were mainly of thermophilic nature, while those particular to the heat^S^ variety were mesophilic. The number of OTUs for *Beggiatoa* and *Pantoea* varies rapidly between the heat^T^ and heat^S^ varieties.

Hone et al. (2021) showed that the microbial diversity and abundance of wheat seed microbiome would vary between the drought-tolerant and -susceptible lines under drought and rainfed conditions, in which the lines subjected to drought had a greater Shannon index, implying more diversity. Jochum et al. (2019) also recognized the improved alpha and beta diversities of wheat microbiome after successive drought stress. In contrast, there are other studies that suggest a decline in the diversity of sorghum and wheat microbiomes (Le Cocq et al., 2017; Safin et al., 2018), and the Shannon diversity of modern wheat cultivars was higher than that of primitive landraces (Gholizadeh et al., 2022). However, in this study, the H–index of heat^T^ V2 was higher than V1 and V3 in the metagenomic composition, while in the case of culturables, the H–index was found to be very low in V2, moderate in V3, and the highest in heat^S^ V1. The diversity of shoot endophytic communities (Sobs and Chao 1) was relatively less than that of the root communities (Liu et al., 2017). In addition, it is believed that the reduction in Shannon index and evenness denotes a lesser diversity, while more species richness and greater diversity are believed to be crucial for managing stress conditions due to higher metabolic rates (Nautiyal and Dion, 2008). Likewise, this study demonstrated high evenness in the heat^S^ variety compared to the other heat^T^ varieties. Hence, the diversity indices as a whole show that there is a greater abundance and diversity in V2 followed by V1 and V3. This study also depicts that the culturable diversity is very high in heat^S^ V1 than in heat^T^ varieties, which could be related to the selection pressure on microbial communities under stress conditions and also due to selective isolation practices.

## 5 Conclusion

To the best of our knowledge, this study is the first to demonstrate the microbial community composition of wheat varieties with variable heat sensitivity by employing both culturable and unculturable approaches. The findings have significantly shown that there is a remarkable variation in the culturable as well as unculturable microbial abundance and diversity. Even though the population and diversity of culturable bacteria are higher in the heat^S^ variety, the preferential demand for survival under heat-stressed conditions contributes to the lesser diversity of heat^T^ varieties. Moreover, a notable number of unculturable bacteria that are thermophilic by nature, such as *Thermomicrobium*, *Acetivibrio*, *Methylophaga*, *Fervidobacterium*, and *Thermotoga*, are associated with the tolerant varieties that could impart resilience to the plants. Furthermore, the heat sensitivity variable varieties from different agro-climatic zones can be explored to a greater extent to better understand the interaction of microbial communities with the plant, their composition, and the mechanisms by which they stipulate tolerance to heat stress. In the future, manipulating the crops’ efficiency through microbiome approaches would result in phenomenal changes to sustainable agriculture under changing climatic conditions.

## Data availability statement

The original contributions presented in the study are publicly available at NCBI for 16S rRNA gene sequences of Wheat Seed Endophytic Bacteria under accession numbers OP782593 - OP782627 and Metagenome data under BioProject PRJNA944920.

## Author contributions

AK: Methodology, Investigation, Data curation, and Writing—Original draft preparation. AS: Conceptualization, Funding acquisition, Methodology, and Writing—Reviewing and Editing. PS: Visualization and Validation. PS: Wheat seed resources. SG and DP: Operation support. All authors contributed to the article and approved the submitted version.
