# Supplementary material for: Seed endophytic bacterial profiling from wheat varieties of contrasting heat sensitivity
Source: Front Plant Sci. 2023 Apr 6;14:1101818. doi: 10.3389/fpls.2023.1101818 (PMC10117849; doi:10.3389/fpls.2023.1101818)
Supplement: Supplementary file 1 [file DataSheet_1.docx]

***Supplementary Material***

**Supplementary Table 1. Metagenome sequence data for MG-RAST analysis**

|  | **Seeds of Wheat Varieties** | | |
| --- | --- | --- | --- |
| **Sample ID** | **GW322 (V1)**  **Heat^S^** | **HD3298 (V2)**  **Heat^T^** | **HD3271 (V3)**  **Heat^T^** |
| Total no. of sequences | 11414000 | 9993617 | 9056645 |
| Total no. of basepairs | 2562025112 | 2317493646 | 2097209168 |
| Average length of sequences (bp) | 224 | 232 | 232 |
| Sequences failed to pass QC | 2076659 (18.19%) | 1373540 (13.74%) | 1228323 (13.56%) |
| Artificial duplicate reads | 1953345 | 1264385 | 1124474 |
| Sequences with predicted feature | 8182346 (71.69%) | 7563822 (75.69%) | 6900020 (76.19%) |
| Sequences containing rRNA genes | 125049 (1.53%) | 100580 (1.33%) | 93260 (1.35%) |
| Sequences contained predicted proteins with known function | 2788179 (34.03%) | 2479159 (32.73%) | 2261082 (32.77%) |
| Sequences contained predicted proteins with unknown function | 5269118 (64.40%) | 4984083 (65.89%) | 4545678 (65.88%) |

**Supplementary Table 2. Enumeration of culturable wheat seed endophytic bacteria (WSEB)**

| **Bacterial population* in seeds of wheat varieties** | | | |
| --- | --- | --- | --- |
| **Growth media** | **GW322**  **Heat^S^** | **HD3298**  **Heat^T^** | **HD3271**  **Heat^T^** |
| TSA | 14×10^2^±151 | 5.5×10^2^±132 | 9×10^2^±136 |
| WSA | 15×10^2^±68 | 7.5×10^2^±72 | 10×10^2^±107 |
| NA | 57×10^2^±148 | 12×10^2^±123 | 12×10^2^±167 |
| KBA | 22.5×10^2^±89 | 16×10^2^±78 | 11×10^2^±69 |
| Selected morphotypes | 19 | 12 | 13 |

*****cfu g^–1^. Values are the mean of three replications ± SE.

**Supplementary Table 3. Growth of WSEB isolates (No.) at different growth conditions**

| **Level of growth** | | **Optimum growth**  **(++++)** | | | **Slightly less growth**  **(+++)** | | | **Minimum growth**  **(+)** | | | **No growth**  **(-)** | | |
| --- | --- | --- | --- | --- | --- | --- | --- | --- | --- | --- | --- | --- | --- |
| **Growth conditions** | | **V1** | **V2** | **V3** | **V1** | **V2** | **V3** | **V1** | **V2** | **V3** | **V1** | **V2** | **V3** |
| **Total no. of isolates** | | 19 | 12 | 13 | - | - | - | - | - | - | - | - | - |
| **Temperature**  **(°C)** | **35** | 19 | 12 | 13 | - | - | - | - | - | - | - | - | - |
|  | **40** | 17 | 12 | 13 | 2 | - | - | - | - | - | - | - | - |
|  | **45** | 15 | 12 | 13 | 1 | - | - | 3 | - | - | - | - | - |
|  | **50** | 11 | 10 | 9 | 3 | 2 | 2 | 4 | - | 1 | 1 | - | 1 |
|  | **55** | 9 | 8 | 8 | 2 | 4 | 4 | 4 | - | - | 4 | - | 1 |
| **Salt (%)** | **2.5** | 19 | 12 | 13 | - | - | - | - | - | - | - | - | - |
|  | **5** | 12 | 4 | 4 | 7 | 6 | 7 | - | 2 | 2 | - | - | - |
|  | **10** | 3 | 1 | 1 | 11 | 3 | 3 | 5 | 5 | 6 | - | 3 | 3 |
|  | **15** | - | - | - | - | 1 | 1 | - | 2 | 2 | 19 | 9 | 10 |
| **pH** | **5.0** | 15 | 8 | 8 | 4 | - | - | - | 4 | 5 | - | - | - |
|  | **7.0** | 19 | 12 | 10 | - | - | 3 | - | - | - | - | - | - |
|  | **9.0** | 14 | 6 | 4 | 5 | 2 | 1 | 0 | 4 | 3 | - | - | 5 |
| **Moisture stress (PEG 6000)** | **-0.05 MPa** | 15 | 7 | 6 | 4 | 1 | 2 | 0 | 4 | 5 | - | - | - |
|  | **-0.15 MPa** | - | - | 2 | 9 | 1 | 2 | 10 | 7 | 4 | - | 4 | 5 |

*Values shown here are the number of isolates displaying growth or no growth at the specified conditions. (PEG 6000 – Polyethylene Glycol 6000, MPa – Mega Pascal)

**Supplementary Table 4. Qualitative estimation of PGP traits of the selected heat^T^ WSEB isolates**

| **WSEB No.** | **Plant Growth Promoting Traits** | | | | | | |
| --- | --- | --- | --- | --- | --- | --- | --- |
|  | **IAA** | **N** | **P** | **K** | **ZnO** | **ZnP** | **Sid** |
| S1 | ++ | - | - | - | - | - | - |
| S2 | - | - | + | - | - | + | - |
| S3 | +++ | + | + | - | - | - | - |
| S4 | +++ | - | + | - | - | + | - |
| S5 | ++ | + | + | + | - | + | +­ |
| S6 | ++++ | + | + | - | - | ­ | - |
| S7 | ++++ | - | + | - | - | - | - |
| S8 | +++ | - | + | - | - | ­ | - |
| S9 | ++++ | - | - | - | - | - | - |
| S10 | ++++ | - | + | - | - | - | - |
| S11 | ++ | + | + | + | - | + | ­+ |
| S12 | ++++ | + | + | + | - | + | ­+ |
| S13 | ++ | + | ­ | + | - | - | - |
| S14 | ++ | - | + | - | - | - | - |
| S15 | ++++ | - | + | - | - | - | - |
| S16 | +++ | - | + | - | - | + | - |
| S17 | ++++ | + | - | - | - | - | - |
| S18 | ++ |  | + | - | - | ­ | - |
| S19 | ++ | + | + | - | + | + | - |
| S20 | + | + | + | - | - | + | - |
| S21 | + | + | - | - | - | - | - |
| S22 | +++ | - | - | - | - | - | - |
| S23 | ++ | + | + | - | + | + | - |
| S24 | +++ | + | - | + | + | - | ­+ |
| S25 | - | - | - | - | - | + | - |
| S26 | ++++ | - | - | - | - | - | - |
| S27 | +++ | + | + | - | ++ | - | ­+ |
| S28 | ++ | + | + | - | + | + | ­+ |
| S29 | ++ | - | - | - | - | - | - |
| S30 | ++++ | + | + | - | ++ | - | ­+ |
| S31 | +++ | - | - | - | - | + | - |
| S32 | ++ | - | - | - | - | - | - |
| S33 | ++++ | - | - | - | - | - | - |
| S34 | +++ | - | - | - | - | - | - |
| S35 | + | - | - | - | - | - | - |

IAA – Indole Acetic Acid, N – Nitrogen fixation, P- Phosphorus solubilisation, K – Potassium solubilisation, Sid – Siderophore production, ZnO and ZnP – Zinc oxide and Zinc phosphate solubilisation.+– positive activity, - – negative activity

**Supplementary Table 5. WSEB isolates (Nos.) from wheat varieties having PGP traits**

| **PGP traits** | **Seeds of Wheat Varieties** | | | **Total** |
| --- | --- | --- | --- | --- |
|  | **GW322**  **Heat^S^** | **HD3298**  **Heat^T^** | **HD3271**  **Heat^T^** |  |
| IAA | 10 | 12 | 11 | 33 |
| N | 4 | 7 | 4 | 15 |
| P | 9 | 8 | 3 | 20 |
| K | 2 | 2 | 1 | 5 |
| ZnO | 0 | 2 | 4 | 6 |
| ZnP | 4 | 5 | 3 | 12 |
| Sid | 2 | 1 | 4 | 7 |

IAA – Indole Acetic Acid, N – Nitrogen fixation, P- Phosphorus solubilisation, K – Potassium solubilisation, Sid – Siderophore production, ZnO and ZnP – Zinc oxide and Zinc phosphate solubilisation.

**Supplementary Table 6. Identification of potential heat^T^ WSEB isolates by 16S rRNA gene sequencing**

| **Variety** | **WSEB Isolate** | **Identified as** | **Percent identity (%)** | **NCBI Accession number** | **Type strain accession number** |
| --- | --- | --- | --- | --- | --- |
| **V1**  **GW322**  **Heat^S^** | S1 | *Bacillus spizizenii* | 99.93 | OP782593 | DSM 15029 |
|  | S2 | *Pantoeaagglomerans* | 98.13 | OP782594 | DSM3493 |
|  | S3 | *Bacillus stratosphericus* | 99.93 | OP782595 | JCM 13349 |
|  | S4 | *Alcaligenesfaecalis* | 97.13 | OP782596 | ATCC 8750 |
|  | S5 | *Priestiaendophytica* | 99.59 | OP782597 | CIP 106778 |
|  | S6 | *Bacillus haynesii* | 97.59 | OP782598 | NRRL B-41327 |
|  | S7 | *Streptomyces lonarensis* | 97.93 | OP782599 | NCL 716 |
|  | S8 | *Bacillus aerius* | 97.14 | OP782600 | JCM 13348 |
|  | S9 | *Bacillus subtilis* | 97.63 | OP782601 | ATCC 6051 |
|  | S10 | *Stenotrophomonasrhizophila* | 97.65 | OP782602 | DSM 14405 |
|  | S11 | *Staphylococcus warneri* | 98.33 | OP782603 | ATCC 27836 |
| **V2**  **HD3298**  **Heat^T^** | S12 | *Bacillus haynesii* | 98.25 | OP782604 | NRRL B-41327 |
|  | S13 | *Stenotrophomonasrhizophila* | 98.14 | OP782605 | DSM 14405 |
|  | S14 | *Enterobacter hormaechei* | 99.11 | OP782606 | ATCC 49162 |
|  | S15 | *Stenotrophomonaspavanii* | 98.87 | OP782607 | DSM 25135 |
|  | S16 | *Bacillus stratosphericus* | 98.24 | OP782608 | JCM 13349 |
|  | S17 | *Priestiaendophytica* | 99.79 | OP782609 | CIP 106778 |
|  | S18 | *Bacillus aerius* | 97.79 | OP782610 | JCM 13348 |
|  | S19 | *Bacillus subtilis* | 97.89 | OP782611 | ATCC 6051 |
|  | S20 | *Pseudomonas plecoglossicida* | 97.89 | OP782612 | ATCC 700383 |
|  | S21 | *Bacillus spizizenii* | 97.04 | OP782613 | DSM 15029 |
|  | S22 | *Stenotrophomonasrhizophila* | 98.21 | OP782614 | DSM 14405 |
|  | S23 | *Bacillus aerius* | 99.09 | OP782615 | JCM 13348 |
| **V3**  **HD3271**  **Heat^T^** | S24 | *Staphylococcus warneri* | 99.86 | OP782616 | ATCC 27836 |
|  | S25 | *Bacillus subtilis* | 99.30 | OP782617 | ATCC 6051 |
|  | S26 | *Bacillus inaquosorum* | 98.39 | OP782618 | KCTC 13429 |
|  | S27 | *Priestiaendophytica* | 98.28 | OP782619 | CIP:106778 |
|  | S28 | *Bacillus aerius* | 99.86 | OP782620 | JCM 13348 |
|  | S29 | *Stenotrophomonasrhizophila* | 97.41 | OP782621 | DSM 14405 |
|  | S30 | *Brachybacteriumparaconglomeratum* | 99.72 | OP782622 | ATCC 51843 |
|  | S31 | *Bacillus aerius* | 97.44 | OP782623 | JCM 13348 |
|  | S32 | *Stenotrophomonastumulicola* | 97.04 | OP782624 | JCM 30961 |
|  | S33 | *Bacillus haynesii* | 97.81 | OP782625 | NRRL B-41327 |
|  | S34 | *Pseudomonas plecoglossicida* | 98.37 | OP782626 | ATCC 700383 |
|  | S35 | *Bacillus subtilis* | 97.67 | OP782627 | ATCC 6051 |

**Supplementary Table 7. Diversity indices of the culturable and unculturable bacterial OTUs**

| **Alpha Diversity indices** | **Unculturable** | | | **Culturable** | | |
| --- | --- | --- | --- | --- | --- | --- |
|  | **GW322**  **Heat^S^** | **HD3298**  **Heat^T^** | **HD3271**  **Heat^T^** | **GW322**  **Heat^S^** | **HD3298**  **Heat^T^** | **HD3271**  **Heat^T^** |
| Taxa_S | 418 | 450 | 410 | 7 | 5 | 6 |
| Individuals | 17281 | 20692 | 16849 | 11 | 12 | 12 |
| Dominance_D | 0.04838 | 0.0512 | 0.0481 | 0.2562 | 0.3333 | 0.3056 |
| Simpson_1-D | 0.9516 | 0.9488 | 0.9519 | 0.7438 | 0.6667 | 0.6944 |
| Shannon_H | 3.855 | 3.865 | 3.814 | 1.666 | 1.314 | 1.474 |
| Evenness_e^H/S | 0.113 | 0.106 | 0.1105 | 0.7561 | 0.7445 | 0.7274 |
| Brillouin | 3.812 | 3.825 | 3.771 | 1.156 | 0.968 | 1.06 |
| Menhinick | 3.18 | 3.128 | 3.159 | 2.111 | 1.443 | 1.732 |
| Margalef | 42.74 | 45.18 | 42.03 | 2.502 | 1.61 | 2.012 |
| Equitability_J | 0.6387 | 0.6326 | 0.6339 | 0.8563 | 0.8167 | 0.8224 |
| Fisher_alpha | 77.18 | 81.15 | 75.81 | 8.286 | 3.218 | 4.775 |
| Berger-Parker | 0.1316 | 0.1308 | 0.1173 | 0.4545 | 0.5 | 0.5 |
| Chao-1 | 476.9 | 514.7 | 475.1 | 22 | 8 | 9 |

**
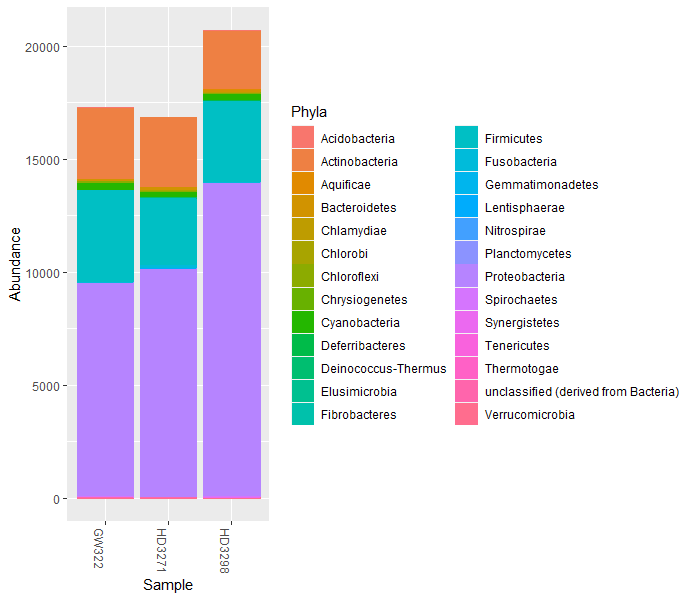
**

**Supplementary Figure 1. Relative abundance of bacterial phyla in heat^S^ (GW322) and heat^T^ (HD3298 & HD3271) wheat varieties**

**
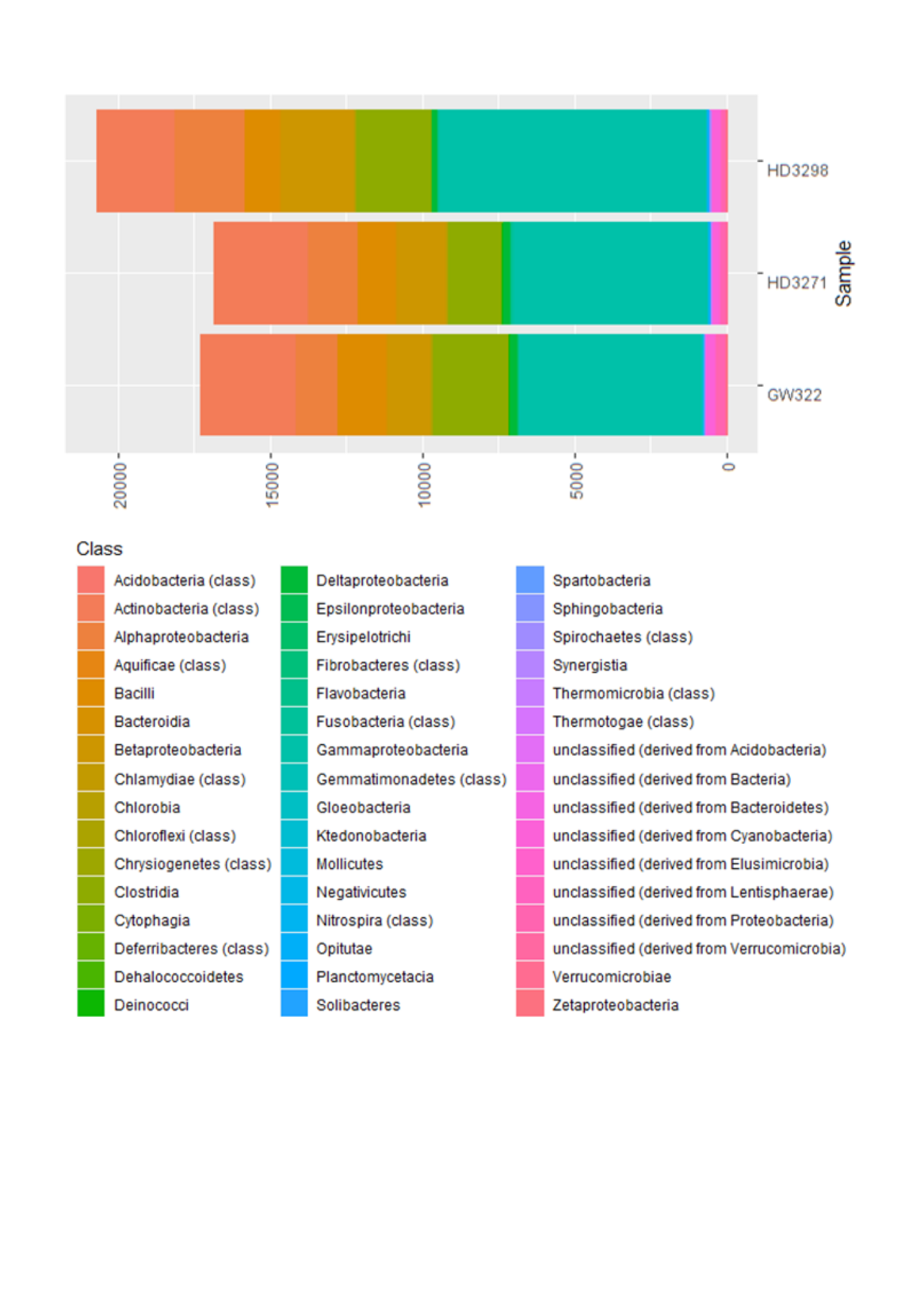
**

**Supplementary Figure 2. Class level distribution of bacterial taxonomy in the metagenome of heat^S^ (GW322) and heat^T^ (HD3298 & HD3271) wheat varieties**


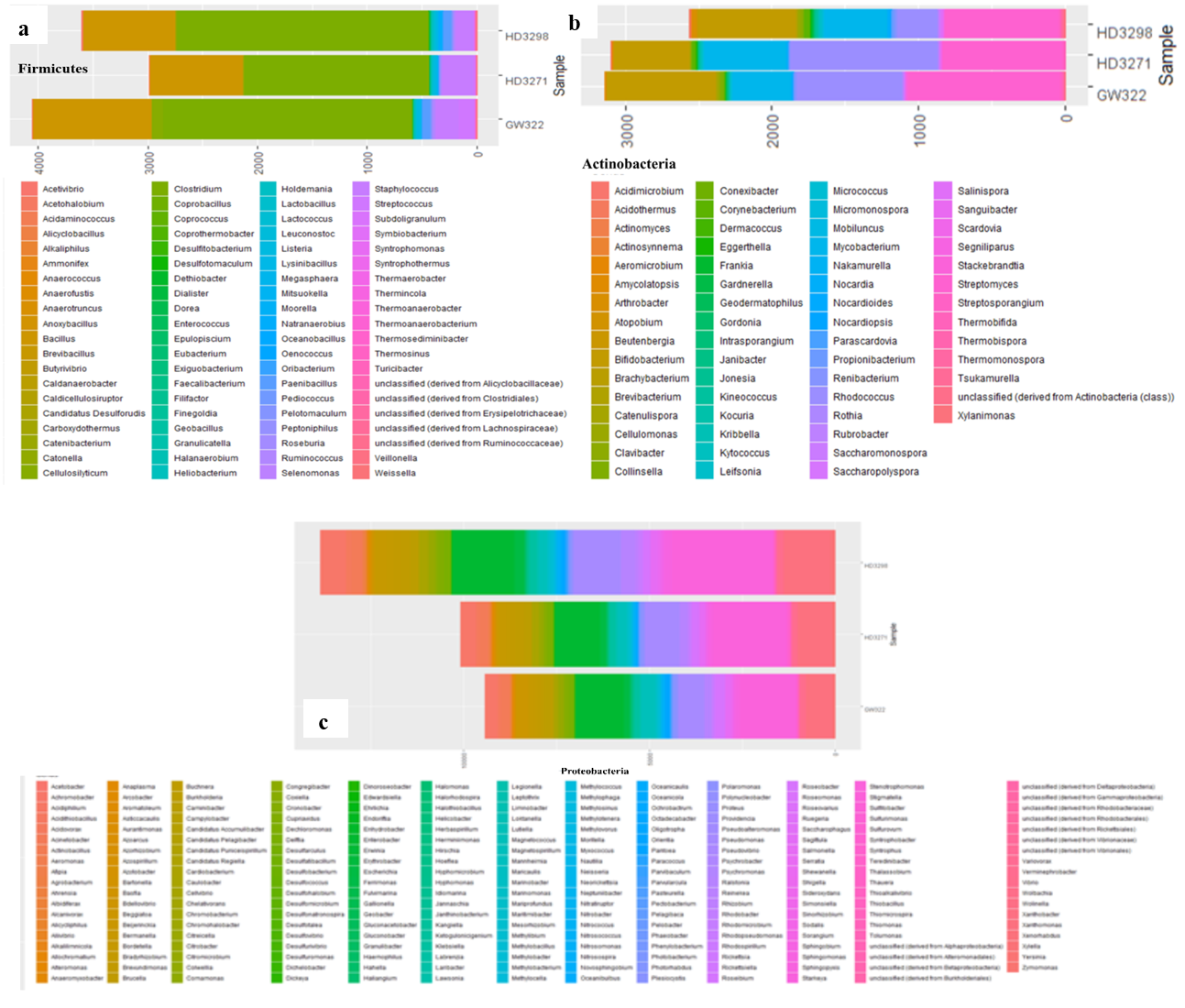


**Supplementary Figure 3. Relative abundance of bacteria in genus level of heat^S^ (GW322) and heat^T^ (HD3298 & HD3271) wheat varieties. a) Genera belonging to phylum Firmicutes, b) Genera belonging to phylum Actinobacteria, c) Genera belonging to phylum Proteobacteria**
